# Screening of TB Actives for Activity against Nontuberculous Mycobacteria Delivers High Hit Rates

**DOI:** 10.3389/fmicb.2017.01539

**Published:** 2017-08-15

**Authors:** Jian Liang Low, Mu-Lu Wu, Dinah Binte Aziz, Benoît Laleu, Thomas Dick

**Affiliations:** ^1^Department of Medicine, Yong Loo Lin School of Medicine, National University of Singapore Singapore, Singapore; ^2^Medicines for Malaria Venture Geneva, Switzerland; ^3^Department of Microbiology and Immunology, Yong Loo Lin School of Medicine, National University of Singapore Singapore; ^4^New Jersey Medical School, Public Health Research Institute, Rutgers, The State University of New Jersey Newark, NJ, United States

**Keywords:** pathogen box, NTM, *Mycobacterium avium*, *Mycobacterium abscessus*, drug screening

## Abstract

The prevalence of lung disease due to infections with nontuberculous mycobacteria (NTM) has been increasing and surpassed tuberculosis (TB) in some countries. Treatment outcomes are often unsatisfactory, highlighting an urgent need for new anti-NTM medications. Although NTM in general do not respond well to TB specific drugs, the similarities between NTM and *Mycobacterium tuberculosis* at the molecular and cell structural level suggest that compound libraries active against TB could be leveraged for NTM drug discovery. Here we tested this hypothesis. The Pathogen Box from the Medicines for Malaria Venture (MMV) is a collection of 400 diverse drug-like compounds, among which 129 are known to be active against *M. tuberculosis*. By screening this compound collection against two NTM species, *Mycobacterium abscessus* and *Mycobacterium avium*, we showed that indeed the hit rates for NTM among TB active compounds is significantly higher compared to compounds that are not active against TB. MIC/dose response confirmation identified 10 top hits. Bactericidal activity determination demonstrated attractive potency for a subset of the confirmed hits. *In vivo* pharmacokinetic profiling showed that some of the compounds present reasonable starting points for medicinal chemistry programs. Three of the top hits were oxazolidinones, suggesting the potential for repositioning this class of protein synthesis inhibitors to replace linezolid which suffers from low potency. Two hits were inhibitors of the trehalose monomycolate transporter MmpL3, suggesting that this transmembrane protein may be an attractive target for NTM. Other hits are predicted to target a range of functions, including cell division (FtsZ), DNA gyrase (GyrB), dihydrofolate reductase, RNA polymerase and ABC transporters. In conclusion, our study showed that screening TB active compounds for activity against NTM resulted in high hit rates, suggesting that this may be an attractive approach to kick start NTM drug discovery projects. In addition, the work identified a series of novel high value NTM hits with associated candidate targets which can be followed up in hit-to-lead projects for the discovery of new NTM antibiotics.

## Introduction

Nontuberculous mycobacteria (NTM)—relatives of the tuberculosis (TB) causative agent *Mycobacterium tuberculosis*, have recently emerged as a new threat to humans (Griffith and Aksamit, 2016; Stout et al., 2016). These bacteria can cause a wide range of illnesses, including TB-like pulmonary disease, which are difficult to cure (Griffith et al., 2007; Griffith, 2010). Therapeutic regimens for NTM infections vary widely, but in general involve a macrolide (clarithromycin or azithromycin)-based multidrug regimen for at least 12–24 months (Griffith et al., 2007; Brown-Elliott et al., 2012). Even so, treatment outcomes are often disappointing especially for *Mycobacterium abscessus* where inducible resistance to macrolides mediated by *erm*(41) gene is frequently found (Nash et al., 2009; Griffith, 2011; Jarand et al., 2011). To make things worse, a recent study demonstrated person-to-person transmission of *M. abscessus* among cystic fibrosis patients (Bryant et al., 2016), overturning the general belief that NTM infections are always independently acquired by individuals through environmental sources (Falkinham, 2009).

In comparison with TB, the NTM drug pipeline is remarkably empty, calling for an urgent need to develop anti-NTM drugs (Griffith and Aksamit, 2016; Raju et al., 2016). The fact that NTM are in general resistant to most anti-tubercular drugs suggests a drug susceptibility profile distinct from *M. tuberculosis* (Pang et al., 2015; Cowman et al., 2016). However, previous genome studies showed that NTM species e.g., *M. abscessus* and *M. avium* share considerable sequence similarities with *M. tuberculosis* (Li et al., 2005; Ripoll et al., 2009). *M. abscessus* has also been shown to possess conserved molecular strategies for host adaptation and persistence with *M. tuberculosis*, as described in a recent transcriptome study (Miranda-CasoLuengo et al., 2016). These similarities between NTM and *M. tuberculosis* suggest that advances made in TB research over the past decade in terms of both understanding the biology and the generation of chemical matter, may be leveraged for NTM drug discovery (Raju et al., 2016). So far, only one report has used a library of TB active compounds for an NTM screen and successfully identified one promising anti-NTM lead (Dupont et al., 2016). Here, we aimed at testing the hypothesis that screening TB actives would yield high hit rates for NTM and therefore could be considered as an attractive strategy for NTM drug discovery.

The Pathogen Box® library^1^ established by the Medicines for Malaria Venture (MMV) consists of 400 drug-like molecules with activity against various neglected diseases. The compounds have been tested for cytotoxicity and values are within levels considered acceptable for an initial drug discovery program. 129 of the library molecules (including 13 reference compounds) have been shown to be active against *M. tuberculosis*, while the remaining 271 are active against other pathogens, including for instance *Cryptosporidium* and kinetoplastids^1^. This provided an opportunity to compare directly the hit rates for NTM between TB active and “non-TB active”^2^ compound collections. In this study, we employed two clinically significant NTM species—*M. abscessus* [representative of rapidly growing NTM; causing incurable lung disease (Griffith, 2011)] and *M. avium* [slowly growing NTM; most commonly isolated species associated with pulmonary NTM disease (Stout et al., 2016)], and screened the Pathogen Box library against both species.

## Materials and methods

### Compound library and chemicals

For the primary screen, 10 mM stock solutions of 400 Pathogen Box compounds were provided by Medicines for Malaria Venture. For dose response assays, fresh powder for 10 hits were resupplied and each compound was dissolved in 90% DMSO to prepare 10 mM stocks. Clarithromycin and rifampicin (Sigma Aldrich) were dissolved in 90% DMSO and filter sterilized with 0.2 μm PTFE membrane filters (Acrodisc, PALL). The final concentration of DMSO in each assay well was always lower than 1%. Tolerance of mycobacteria for 1% DMSO was tested and this concentration of solvent did not affect growth of bacteria (data not shown).

### Bacterial strains and culture conditions

*Mycobacterium abscessus* Bamboo was isolated from the sputum of a patient with amyotrophic lateral sclerosis and bronchiectasis and was provided by Wei Chang Huang, Taichung Veterans General Hospital, Taichung, Taiwan. Whole genome sequencing showed that the strain belongs to *M. abscessus* subsp. *abscessus* and harbors an inactive, clarithromycin sensitive, *erm*(41) C28 sequevar (Yee et al., 2017). *M. avium* 11 was isolated from the bone marrow of a patient with acquired immunodeficiency syndrome with disseminated infection and was provided by Jung-Yien Chien and Po-Ren Hsueh, National Taiwan University Hospital, Taipei. Whole genome sequencing showed that the strain belongs to *M. avium* subsp. *hominissuis* (Yee et al., in press). *M. tuberculosis* H37Rv (ATCC 27294) was purchased from the American Type Culture Collection, Manassas, VA. All mycobacteria were cultivated in complete Middlebrook 7H9 medium (BD Difco) supplemented with 0.2% glycerol (Fisher Scientific), 0.05% Tween 80 (Sigma), and 10% Middlebrook albumin-dextrose-catalase (BD Difco) at 37°C with 15 rpm agitation. *Mycobacterium abscessus* Bamboo and *Mycobacterium avium* 11 solid cultures were grown on complete Middlebrook 7H10 supplemented with 0.5% glycerol and 10% Middlebrook oleic acid-albumin-dextrose-catalase (BD Difco) at 37°C.

### Single point growth inhibition screening assay

The screening assay was carried out as described previously (Aziz et al., 2017). Mid-log phase bacterial cultures were diluted to OD_600_ = 0.1 or 10^7^ CFU/mL. The bacterial suspension was then dispensed into 96 well plates (Corning Costar #CLS3599) containing compounds to give a final volume of 200 μL per well with a final OD_600_ of 0.05 and final compound concentration of 20 μM. The plates were sealed with Breathe-Easy sealing membrane (Sigma-Aldrich) and placed in airtight boxes containing wet paper towels and incubated at 37°C with shaking at 110 rpm for 3 days for *M. abscessus* or 4 days for *M. avium*. Growth was determined by measuring OD_600_ using an Infinite M200 Pro plate reader (Tecan) after manual resuspension of the bacteria in the wells. Percentage growth inhibition was calculated for each well using the untreated control cultures on the same plate as reference. Clarithromycin was used as positive control in each plate. The screen was carried out in two biological replicates. The standard deviations for the data points (% growth inhibition) generated for each compound was ±10%.

### Dose-response growth inhibition and bactericidal assay

Hits were reconfirmed in a dose response assay employing the broth microdilution method as described previously (Aziz et al., 2017). Briefly, the hit compounds were 2-fold serial diluted from 100 to 0.4 μM. Mid-log phase cultures were diluted and dispensed into 96 well plates (final starting inoculum: OD_600_ = 0.05; final compound concentrations: 50–0.2 μM; final volume: 200 μL per well). The plates were incubated as mentioned above. For *M. tuberculosis*, the plate cultures were grown for 7 days. MIC_50_ and MIC_90_ were determined as the concentration that inhibits 50% or 90% of bacterial growth, respectively. Rifampicin was used as the positive control for *M. tuberculosis* while clarithromycin was used for *M. abscessus* and *M. avium*.

Bactericidal activities of the reconfirmed hits were determined for *M. abscessus* and *M. avium* by exposing 10^7^ CFU/mL to their respective MIC_90_ concentration for 3 or 4 days at 37°C, respectively. The treated cultures were then serial diluted and the appropriate dilutions were plated on 7H10 agar and incubated at 37°C. CFU were enumerated after 3 days of incubation for *M. abscessus* and 7 days of incubation for *M. avium*. Fold kill was calculated by comparing CFU after drug treatment with the starting inoculum.

## Results

### Screening TB active compounds against NTM results in significantly higher hit rates as compared to screening non-TB active compounds

**Figure 1:**
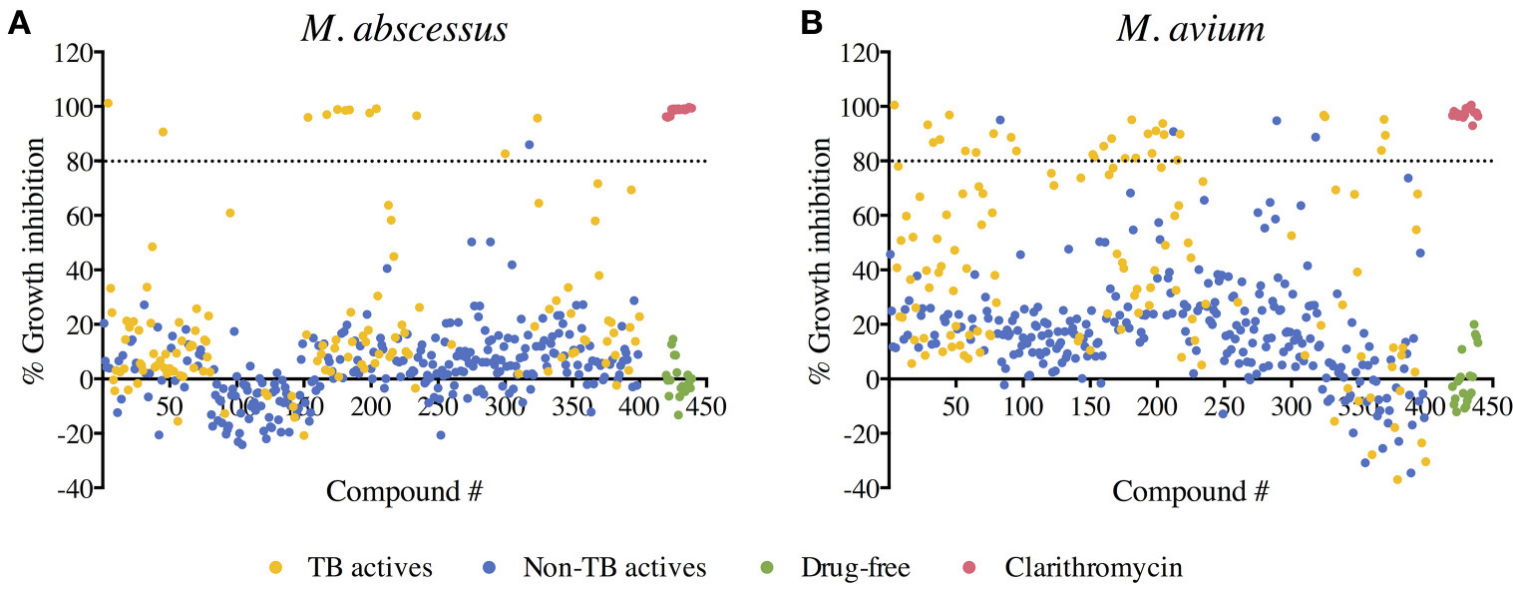
Primary screen: compound—growth inhibition scatter plot for **(A)** *M. abscessus* and **(B)** *M. avium*. A total of 400 compounds from the Pathogen Box were screened for growth inhibition at 20 μM against *M. abscessus* and *M. avium*. Out of the 400 test compounds, 129 are TB actives (shown in yellow) and 271 are non-TB actives (shown in blue). Drug free controls are shown in green and positive drug controls (Clarithromycin) are shown in red. Average % growth inhibition of duplicate data points for each compound is shown. Compounds with equal to—or more than—80% growth inhibition activity (above the black dashed line) were defined as hits.

**Figure 2:**
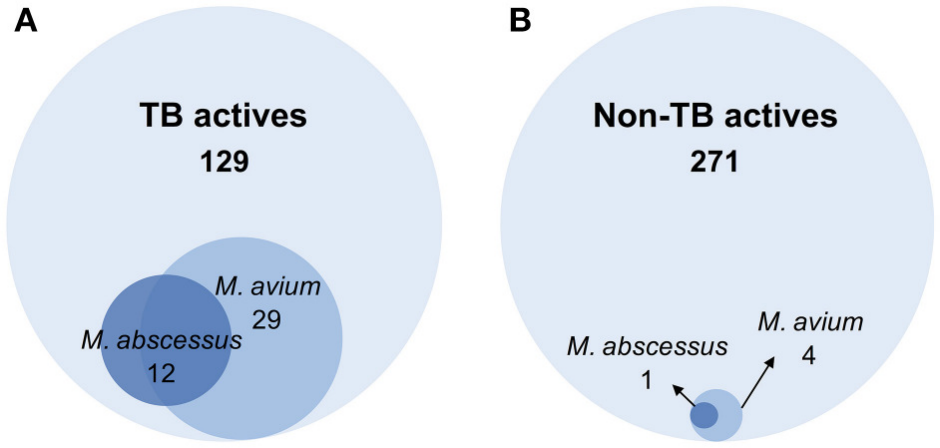
Venn diagrams comparing the number of hits against *M. abscessus* and *M. avium* within **(A)** TB active and **(B)** non-TB active library compounds. **(A)** Out of 129 compounds active against *M. tuberculosis* (TB actives), 12 were active against *M. abscessus* and 29 were active against *M. avium*. **(B)** Among 271 non-TB actives, 1 compound was active against *M. abscessus* and 4 were active against *M. avium*. The hit active against *M. abscessus* also showed activity against *M. avium*.

A primary screen was performed using 20 μM final drug concentration for all 400 Pathogen Box compounds against two clinical strains, *M. abscessus* Bamboo and *M. avium* 11. Compounds which exhibited equal or more than 80% growth inhibition were defined as hits. As shown in Figure 1A, the single point screen resulted in 13 *M. abscessus* hits, all of which were TB actives except one cryptosporidiosis-active compound. This corresponds to a 9.3% hit rate for the 129 TB actives and a hit rate of 0.4% for the 271 non-TB actives. 33 *M. avium* hits were also identified from the initial screen (Figure 1B), out of which 29 were TB actives. This corresponds to a 22.5% hit rate for TB actives and a 1.5% hit rate for non-TB actives. These results show that for both *M. abscessus* and *M. avium* the hit rate was significantly higher for TB actives as compared to non-TB active compounds (Figure 2). In accordance with previous reports (Nessar et al., 2012; van Ingen et al., 2012; Pang et al., 2015) these results also demonstrates the high intrinsic resistance of *M. abscessus* to a variety of drugs: the hit number of *M. abscessus* was only half to one-third of that of *M. avium*. Interestingly, the majority of the *M. abscessus* hits were also active against *M. avium* (Figure 2).

Among the primary hits, nine compounds were double hits, i.e., they were active against both *M. abscessus* and *M. avium*. Consistent with literature, all reference compounds, namely bedaquiline (MMV689758), levofloxacin (MMV687798) and linezolid (MMV687803), expected to have activity against both *M. abscessus* and *M. avium* indeed showed activity (Supplementary Figure S1; Rastogi et al., 1996; Brown-Elliott et al., 2003; Pang et al., 2017). In addition, expected *M. avium*-specific hits included streptomycin (MMV688994), rifampicin (MMV688775), clofazimine (MMV687800), mefloquine (MMV000014) and delamanid (MMV688262) (Supplementary Figure S1). The former two antibiotics are currently recommended for treatment of *M. avium* complex lung disease (Griffith et al., 2007), whereas the latter three have been shown to be active on *M. avium* (Bermudez et al., 1999; Ferro et al., 2016; Krieger et al., 2016) but their potential roles in treatment have not yet been established.

### Several pathogen box hits exhibit attractive bactericidal potency against NTM

With the aim of identifying broad spectrum antimycobacterial compounds, six double hits (excluding the three reference compounds) were selected and fresh solids were used to confirm these hits. Keeping in mind the limited treatment options for the notoriously difficult-to-treat *M. abscessus*, four potent “*M. abscessus*-specific” hits were also included in the hit confirmation. Confirmation of hits was carried out by performing a dose response assay to determine the minimum inhibitory concentration (MIC) against three strains, *M. tuberculosis* H37Rv, *M. abscessus* Bamboo and *M. avium* 11. In Table 1, we report MIC_50_ and MIC_90_ which are the concentrations that inhibit 50 or 90% of growth, respectively, as compared to the untreated control. Clarithromycin is added in Table 1 as a positive control: MIC data for clarithromycin are consistent with earlier reports (Heifets et al., 1992; Bastian et al., 2011). Table 1 also shows that our *M. tuberculosis* MIC_90_ data were consistent with the respective MIC data reported previously by MMV (website http://www.pathogenbox.org/).

**Table 1:** Antimycobacterial activities (μM) of top NTM hits identified in the Pathogen Box screen.

**Compound ID**	***M. tuberculosis***			***M. abscessus***			***M. avium***			Chemical class^*^	Possible target^*^
	**MIC_50_**	**MIC_90_**	**Reported MIC^b^**	**MIC_50_**	**MIC_90_**	**Fold kill at MIC_90_**	**MIC_50_**	**MIC_90_**	**Fold kill at MIC_90_**		
MMV688756	<0.2	1.2	1.2	4.2	25	NK	0.4	4	NK	Oxazolidinone	23S rRNA
MMV688327^a^	0.5	2	0.3	3.8	13	NK	4	35	NK	Oxazolidinone	23S rRNA
MMV688508	<0.2	0.3	0.4	2.2	12	NK	0.6	8	NK	Oxazolidinone	23S rRNA
MMV687146	<0.2	<0.2	<0.024	0.8	2	100	3	9	NK	Indolecarboxamide	MmpL3
MMV688846^a^	0.2	0.6	0.4	0.7	1.5	10	20	>50	NK	Piperidinol	MmpL3
MMV687730	<0.2	<0.2	0.05	4	8.5	10	5	22	NK	Benzimidazole	FtsZ
MMV687812^a^	<0.2	2.2	1.2	6	12^∧^	NK^∧^	25	>50	NK	Aminopyrazinamide	GyrB
MMV675968	<0.2	4	ND	10.5	35	NK	0.2	3	NK	Diaminopyrimidine	DHFR
MMV688845	<0.2	1.7	1.2	1.5	7	10	0.6	3	NK	Phenylalaninamide	RNA polymerase
MMV688844^a^	0.2	2.8	0.4	4.5	11	100	2	6^#^	NK^#^	Piperidine	ABC transporter
clarithromycin	ND	ND	ND	<0.2	0.5	NK	<0.2	0.6	NK	Macrolide	23S rRNA

a *“M. abscessus-specific” hits according to primary screen cut-off*.

b *MIC in M. tuberculosis as reported by MMV (website http://www.pathogenbox.org/)*.

∧ *MMV687812, for M. abscessus 80% growth inhibition was achieved at 12 μM, MIC_90_ could not be reached up to 50 μM*.

# *MMV688844, for M. avium 80% growth inhibition was achieved at 6 μM, MIC_90_ could not be reached up to 50 μM*.

* *See discussion section for references*.

*MIC_50_, MIC_90_ refers to the compound concentration that inhibits 50 and 90% of bacterial growth, respectively*.

*NK, “no kill,” i.e., CFU reduction < 10-fold at MIC_90_; ND, not determined*.

**Figure 3:**
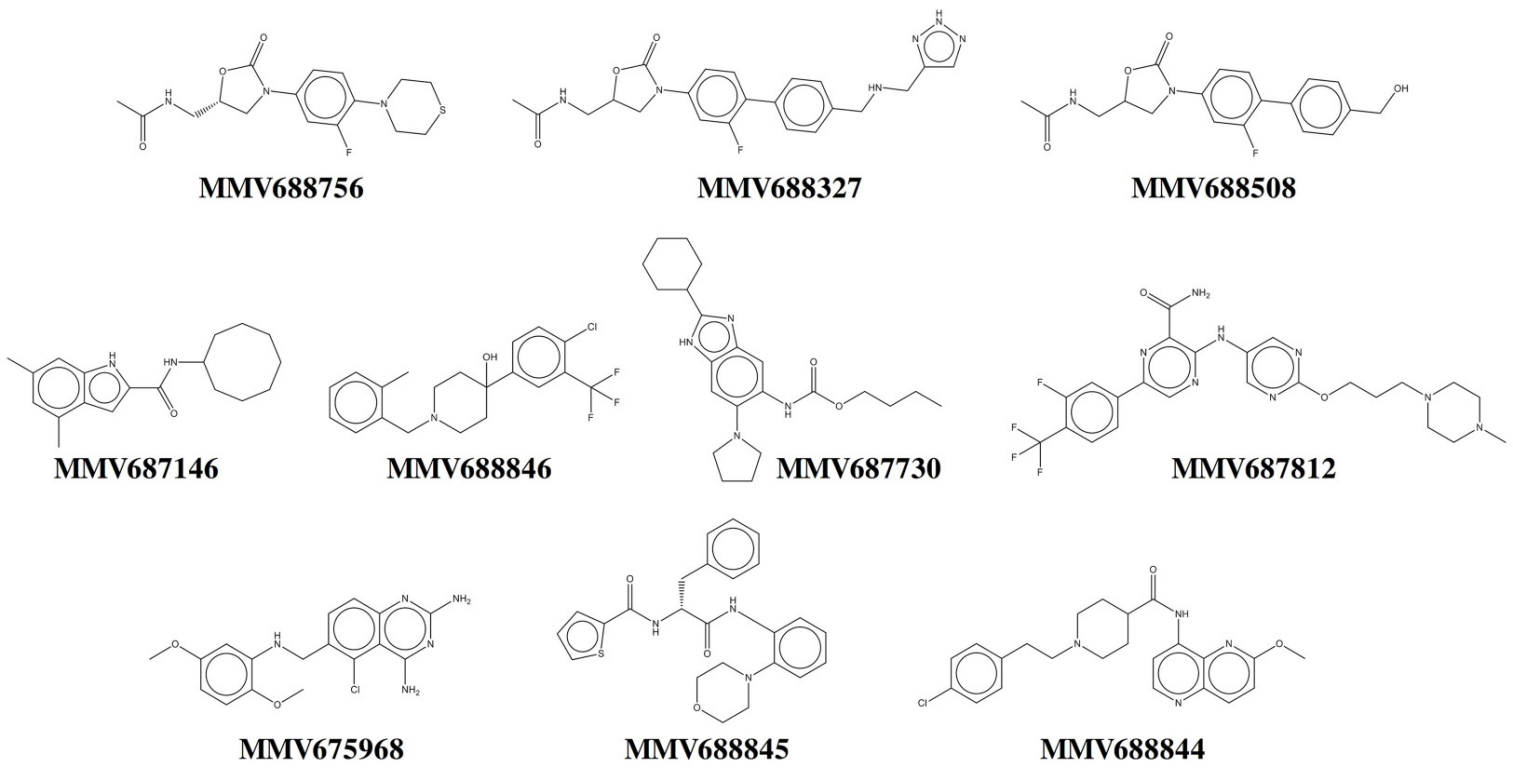
Chemical structures of top NTM hits identified in the Pathogen Box screen.

Our results show that all primary hits were confirmed. These hits belong to a variety of chemical classes including oxazolidinones, indolecarboxamides, and benzimidazoles (Table 1, Figure 3). Two out of the 10 hits, MMV688756 (sutezolid), and MMV688327 (radezolid), are drugs under clinical development for treatment of TB or other infections (Pandit et al., 2012), but neither of these two oxazolidinones have been reported for their anti-NTM properties before.

To further assess the potential of these hits we determined the cidal activity of each compound at its MIC_90_ against *M. abscessus* and *M. avium*. Table 1 shows that several of the hits displayed in addition to low growth inhibitory concentrations, attractive cidal activities. The lack of bactericidal antibiotics in the currently available treatment regimens has been proposed to be one of the reasons for the poor therapeutic outcomes of NTM diseases (Griffith, 2014; Maurer et al., 2014). Thus, a significant cidal activity at MIC_90_ is an attractive property. The concentration required for clarithromycin, the cornerstone in current NTM therapy, to reduce CFU 10-fold is up to 100 times higher than its MIC_90_ for *M. abscessus* (Aziz et al., 2017) and up to 64 times higher than its MIC_90_ for *M. avium* (Heifets et al., 1992).

## Discussion

To test the hypothesis that NTM hit rates may be high for libraries of compounds that are active against *M. tuberculosis*, we screened the Pathogen Box which comprises of 129 TB active and 271 non-TB active compounds against two clinically relevant NTM species, *M. abscessus* and *M. avium*. With a cut-off of 80% inhibition at 20 μM, our primary screen yielded indeed high hit rates of NTM actives (~10% for *M. abscessus* and ~20% for *M. avium*) among the 129 TB active compounds. In comparison, the hit rates for non-TB active compounds were 15–25 fold lower. In TB drug discovery, hit rates for whole cell screens using large (“random”) small molecule libraries against *M. tuberculosis* are often below 1% (Ekins et al., 2013). Similarly for NTM drug discovery, our recent screen of 2700 FDA approved drugs against *M. abscessus* resulted in ~1% hit rate (Aziz et al., 2017). Our results here suggest that screening libraries of TB hits may be an effective approach for generating the substrates for kick-starting NTM drug discovery projects.

*Mycobacterium abscessus* has been shown to exhibit high level of intrinsic resistance against a large number of antibiotics (van Ingen et al., 2010; Cowman et al., 2016). Similarly, we observed from our screen that *M. abscessus* has a lower hit rate when compared to *M. avium*, consistent with the notion that this pathogen is intrinsically more resistant to a broad spectrum of compounds than both *M. avium* and *M. tuberculosis*. Numerous mechanisms have been proposed for the natural resistance of *M. abscessus*, including low cell envelope permeability, efflux and intrabacterial drug metabolism (Ripoll et al., 2009; Nessar et al., 2012; van Ingen et al., 2012; Aziz et al., 2017). From a phylogenetic point of view, the rapid grower *M. abscessus* is more distantly related to *M. tuberculosis* than the slow growing *M. avium* (Mignard and Flandrois, 2008). Comparative genomics studies also revealed that the *M. abscessus* genome not only harbors less orthologues paired with *M. tuberculosis* than *M. avium* (Prasanna and Mehra, 2013), but has acquired a large reservoir of genes horizontally transferred from other soil or water dwellers (Ripoll et al., 2009). An interesting trend suggested by our results is that *M. abscessus* hits represent a subset of *M. avium* hits, i.e., that *M. abscessus* hits appear to display broad spectrum antimycobacterial activity (Figure 2). This suggests the feasibility of developing broad spectrum antimycobacterials to address both NTM as well as TB disease.

Based on dose response determination we selected 10 top hits (6 double hits and 4 *M. abscessus*-specific hits) for further characterization (Tables 1, 2, Figure 3). Most of the top hits had a MIC < 10 μM against either *M. abscessus* or *M. avium*. Antibiotics recommended in current NTM treatment (besides clarithromycin/azithromycin) include amikacin, cefoxitin, imipenem and tigecycline for *M. abscessus*, and rifampicin/rifabutin, ethambutol and streptomycin/amikacin for *M. avium* (Griffith et al., 2007; Brown-Elliott et al., 2012). However, most of these drugs exhibit a variable and in general moderate growth inhibitory activity against NTM [e.g., MIC for imipenem on *M. abscessus* is around 27 μM (8 mg/L) (Lavollay et al., 2014)] with no or weak bactericidal activity (Maurer et al., 2014).

**Table 2:** Physicochemical and *in vivo* pharmacokinetic properties of top NTM hits identified in the Pathogen Box screen^a^.

**Compound ID**	**MW**	**cLogP**	**Route/Dose (mg/kg)**	**t_max_ (h)**	**C^0^/C_max_(ng/mL)**	**AUC_last_(ng·h/mL)**	**AUC_inf_(ng·h/mL)**	**Cl (mL/min/kg)**	**V_ss_(L/kg)**	**t_1/2_(h)**	**%F**
MMV688508	358.4	1.91	IV/1	NA	1310	156	156	280	1.59	0.0724	-
			PO/5^#^	0.25	1.16	1.45	NR	NA		NR	<1
MMV687146	298.4	4.85	IV/1	NA	2270	798	935	18.2	9.77	14.9	-
			PO/5	0.75	245	456	483	NA		3.48	11
MMV688846	383.8	4.95	IV/1	NA	563	423	464	36.8	14.4	9.78	-
			PO/5	1.5	3.19	18.1	27.5	NA		4.6	<1
MMV687730	384.5	5.00	IV/1	NA	444	108	125	166	15.9	3.34	-
			PO/5	NC (BLQ at all time points)							ND
MMV687812	534.5	2.35	IV/1	NA	265	381	801	20.8	49.6	30.5	-
			PO/5	7	55.3	962	2450	NA		30.5	50
MMV675968	359.8	2.31	IV/1	NA	1080	897	900	18.7	1.31	1.03	-
			PO/5	1	138	504	532	NA		1.7	11
MMV688845	435.5	3.23	IV/1	NA	716	171	174	96.3	4.5	1.28	-
			PO/5	0.375	2.28	3.95	NR	NA		NR	<1
MMV688844	424.9	3.83	IV/1	NA	116	164	167	102	11	1.6	-
			PO/5	NC (BLQ at all time points)							ND

a *Data were obtained by MMV (http://www.pathogenbox.org/). DMPK experiments were carried out in Sprague Dawley Male rats (n = 2 animals), the mean PK parameters are shown in the table. IV formulation: 10% DMSO, 40% PEG 400 and 2% Tween 80 in Milli-Q water; PO formulation: 0.5% HPMC, 0.5% benzyl alcohol and 0.4% Tween 80 in Milli-Q water*.

*AUC_last_, area under the curve from time 0 to the last time point; AUC_inf_, area under the curve from time 0 extrapolated to infinite time; V_ss_, apparent volume of distribution at steady state*.

*BLQ, below the limit of quantification (< 1 ng/mL); NA, not applicable; ND, not determined; NC, not calculable; NR, not reportable due to inadequate elimination phase*.

# *Data for n = 1 animal*.

Three of the 10 top hits are oxazolidinone derivatives likely targeting the ribosome: sutezolid (MMV688756), radezolid (MMV688327) and a synthesis intermediate of radezolid (MMV688508). Linezolid, being the first FDA approved oxazolidinone, has some activity against NTM but suffers from low potency (MIC_90_ is 36 μM for *M. abscessus* and 22 μM for *M. avium*) (Brown-Elliott et al., 2003; Cavusoglu et al., 2007; Aziz et al., 2017). Despite its low potency, reports of successful therapy employing multidrug regimens containing linezolid indicate the clinical usefulness of this drug class for the treatment of NTM disease (Nannini et al., 2002; Lee et al., 2010). The identification of more potent oxazolidinone derivatives suggests that repositioning of linezolid for the treatment of NTM lung infections may be an attractive approach. It is interesting to note that as in *M. tuberculosis* (Williams et al., 2009; Alffenaar et al., 2011), sutezolid is also more active than linezolid. Furthermore, activity of the oxazolidinones as well as the macrolides against NTM suggest that protein synthesis is an attractive target for NTM.

Two hits, MMV687146 (an indole-2-carboxamide) and MMV688846 (a piperdinol), have been reported to target the trehalose monomycolate transporter MmpL3 and disrupt mycolic acid synthesis in mycobacteria (Lun et al., 2013; Dupont et al., 2016). In recent years, with the discovery of several new TB drug leads, such as SQ109, BM212 and AU1235, MmpL3 has become an attractive novel target for tuberculosis (Li et al., 2016b). The *M. tuberculosis* MmpL3 inhibitor SQ109 (Tahlan et al., 2012) has a MIC of 4–16 mg/L (12–48 μM) for *M. abscessus* and *M. avium* (Sacksteder et al., 2012). Previously, anti-*M. abscessus* activity of MMV688846 (also known as GSK1985270A or PIPD1) has been demonstrated by Dupont et al. (2016). Here, by reporting the excellent anti-*M. abscessus* activity of another MmpL3 inhibitor (MMV687146) with a different scaffold, we show that similar to *M. tuberculosis*, MmpL3 may be an appealing target for *M. abscessus* due to its potential essentiality in mycobacterial species (Li et al., 2016b). Nevertheless, both MmpL3 inhibitors from our top hits are less active against *M. avium* than *M. tuberculosis* and *M. abscessus*, suggesting that there might be some natural polymorphisms in the *M. avium mmpL3* gene which are responsible for its higher intrinsic resistance.

MMV687730 is a benzimidazole that has been reported to inhibit the assembly of FtsZ in *M. tuberculosis* by enhancing the GTPase activity and destabilizing FtsZ polymer (Kumar et al., 2011; Li et al., 2016a). FtsZ is essential for growth in NTM species (Dziadek et al., 2003), and hence represents a new NTM target for therapeutic intervention.

MMV687812 is an aminopyrazinamide which binds specifically to mycobacterial GyrB at its ATPase domain (Shirude et al., 2013). Previously, another GyrB inhibitor VXc-486 (an aminobenzimidazole) has been shown to inhibit the growth of *M. abscessus* and *M. avium* (Locher et al., 2015). These findings warrant further development of GyrB inhibitors for NTM.

MMV675968, an anti-cryptosporidiosis compound that inhibits dihydrofolate reductase in *Pneumocystis carinii* and *Toxoplasma gondii* (Rosowsky et al., 1994), turned out to be also active against *M. tuberculosis*, in addition to NTM.

The two novel NTM actives MMV688845 (GSK1729177A) and MMV688844 (TCMDC-143649) were originally identified as non-cytotoxic *M. tuberculosis* hits in GSK whole cell screens (Ballell et al., 2013; Rebollo-Lopez et al., 2015). Recently, an analog of MMV688845 has been shown to target RNA polymerase in *M. tuberculosis* via a binding site different from rifampicin (Ebright et al., 2015; Lin et al., 2017). The TB drug rifampicin itself has a very high MIC (37 μM or higher) for *M. abscessus* therefore it is not in use against this NTM pathogen (Aziz et al., 2017). MMV688844 has been predicted, based on *in silico* analyses, to target ABC transporters (Rv0194) in *M. tuberculosis* (Rebollo-Lopez et al., 2015).

It is important to note that most of the possible targets discussed in Table 1 are derived from work on *M. tuberculosis* and need to be confirmed experimentally in NTM.

To assess the lead potential for the top NTM active hits, physicochemical properties and *in vivo* (rat) pharmacokinetic (PK) profiles were determined (http://www.pathogenbox.org/) and are summarized in Table 2. Table 2 shows that almost all hits displayed favorable molecular weight (< 500 g/mol) and hydrophobicity (cLogP < 5) properties. For MMV688756 (sutezolid) and MMV688327 (radezolid) the pharmacokinetic properties have been described previously (Lemaire et al., 2010; Wallis et al., 2011; Moore et al., 2012). MMV687146, and MMV675968 at a low dose of 5 mg/kg exhibit promising plasma exposure following oral administration, relative to their respective MICs against *M. abscessus* or *M. avium*. MMV688846 shows reasonable exposure at 1 mg/kg, suggesting that medicinal chemistry efforts focusing on improving oral bioavailability could result in successful lead generation. MMV687812 showed favorable PK properties which could be leveraged with more potent analogs. For all other hits, structure-activity-relationship studies with a limited number of analogs can be performed to determine whether adequate PK-PD targets can be achieved. Taken together, the confirmed NTM hits identified in this work constitute valuable starting points for medicinal chemistry programs whereby potency and physiochemical properties can be optimized to achieve appropriate PK-PD profiles.

In conclusion, our study showed that screening collections of TB active compounds against NTM resulted in higher hit rates as compared to screening “random” libraries of non-TB active molecules. Thus, screening chemical matter generated for TB drug discovery over the past decade represents an attractive strategy for NTM drug discovery. In addition, screening the Pathogen Box delivered a series of novel NTM hits with attractive potencies, characterized *in vivo* pharmacokinetic properties and associated candidate targets. Currently we are confirming activity of the hits against a collection of clinical isolates (Aziz et al., 2017) and are initiating a repositioning program for oxazolidinones and hit-to-lead projects with concurrent target identification / confirmation for the other top hits identified in this work.

## Author contributions

TD conceived the project and designed the strategy. JL, MW, and DA carried out the experiments. JL, MW, BL, and TD analyzed the data and wrote the manuscript.

### Conflict of interest statement

The authors declare that the research was conducted in the absence of any commercial or financial relationships that could be construed as a potential conflict of interest.

## Footnotes

^1^http://www.pathogenbox.org/

^2^“non-TB active compounds”: Pathogen Box compounds that have not been previously reported to be active against TB.
