# Supplementary material for: Screening of TB Actives for Activity against Nontuberculous Mycobacteria Delivers High Hit Rates
Source: Front Microbiol. 2017 Aug 15;8:1539. doi: 10.3389/fmicb.2017.01539 (PMC5559473; doi:10.3389/fmicb.2017.01539)
Supplement: Supplementary file 1 [file DataSheet1.PDF]

Supplementary Material

Screening of TB actives for activity against nontuberculous mycobacteria delivers high hit rates

Jian Liang Low[#], Mu-Lu Wu[#], Dinah Binte Aziz, Benoît Laleu, Thomas Dick*

*** Correspondence:** Thomas Dick: td367@njms.rutgers.edu

[#] Both authors contributed equally to the work.

1 Supplementary Figure

Compound ID	Disease Set	Trivial Name	% Inhibition	
			M. abscessus	***M. avium***
**1) Double hits**				
MMV689758	Reference compounds	Bedaquiline	>=80%	>=80%
MMV688756	Tuberculosis	Sutezolid	>=80%	>=80%
MMV687803	Reference compounds	Linezolid	>=80%	>=80%
MMV688508	Tuberculosis		>=80%	>=80%
MMV687730	Tuberculosis		>=80%	>=80%
MMV688845	Tuberculosis		>=80%	>=80%
MMV687146	Tuberculosis		>=80%	>=80%
MMV675968	Cryptosporidiosis		>=80%	>=80%
MMV687798	Reference compounds	Levofloxacin	>=80%	>=80%
**2) *M. abscessus* specific hits**				
MMV688327	Tuberculosis	Radezolid	>=80%	60-79%
MMV688846	Tuberculosis		>=80%	40-59%
MMV688844	Tuberculosis		>=80%	60-79%
MMV687812	Tuberculosis		>=80%	40-59%
**3) *M. avium* specific hits**				
MMV676395	Tuberculosis		<=39%	>=80%
MMV661713	Tuberculosis		<=39%	>=80%
MMV676383	Tuberculosis		<=39%	>=80%
MMV676377	Tuberculosis		<=39%	>=80%
MMV063404	Tuberculosis		<=39%	>=80%
MMV461553	Tuberculosis		<=39%	>=80%
MMV000062	Reference compounds	Pentamidine	<=39%	>=80%
MMV012074	Tuberculosis		<=39%	>=80%
MMV687800	Reference compounds	Clofazimine	60-79%	>=80%
MMV000014	Reference compounds	Mefloquine	<=39%	>=80%
MMV688994	Reference compounds	Streptomycin	<=39%	>=80%
MMV687145	Tuberculosis		<=39%	>=80%
MMV054312	Tuberculosis		<=39%	>=80%
MMV687703	Tuberculosis		<=39%	>=80%
MMV687188	Tuberculosis		<=39%	>=80%
MMV687696	Tuberculosis		<=39%	>=80%
MMV688179	Kinetoplastids		40-59%	>=80%
MMV688262	Tuberculosis	Delamanid	40-59%	>=80%
MMV687807	Tuberculosis		40-59%	>=80%
MMV688271	Kinetoplastids		40-59%	>=80%
MMV688775	Reference compounds	Rifampicin	60-79%	>=80%
MMV687729	Tuberculosis		40-59%	>=80%
MMV687813	Tuberculosis		60-79%	>=80%
MMV153413	Tuberculosis		<=39%	>=80%

Inhibition
>=80%
60-79%
40-59%
<=39%

Supplementary Figure 1. List of hits from the single point screen. A heat map is shown denoting the activity of each hit against *M. abscessus* and *M. avium*: >=80% growth inhibition (dark orange); 60-79% growth inhibition (light orange); 40-59% growth inhibition (beige); 0-39% inhibition (white). 'Disease set' is as provided by MMV (website http://www.pathogenbox.org/).
